# A Visual Framework for Classifying Determinants of Cell Size

**DOI:** 10.1016/j.celrep.2018.11.087

**Published:** 2018-12-18

**Authors:** Felix Jonas, Ilya Soifer, Naama Barkai

**Affiliations:** 1 Department of Molecular Genetics, Weizmann Institute of Science, Rehovot 76100, Israel; 2 Calico Labs, South San Francisco, CA 94080, USA

**Keywords:** cell size, dynamical systems, single cell, cell cycle, S. cerevisiae

## Abstract

Cells control their size by coordinating cell cycle progression with volume growth. Size control is typically studied at specific cell cycle transitions that are delayed or accelerated depending on size. This focus is well suited for revealing mechanisms acting at these transitions, but neglects the dynamics in other cell cycle phases, and is therefore inherently limited for studying how the characteristic cell size is determined. We address this limitation through a formalism that intuitively visualizes the characteristic size emerging from integrated cell cycle dynamics of individual cells. Applying this formalism to budding yeast, we describe the contributions of the un-budded (G1) and budded (S-G2-M) phase to size adjustments following environmental or genetic perturbations. We show that although the budded phase can be perturbed with little consequences for G1 dynamics, perturbations in G1 propagate to the budded phase. Our study provides an integrated view on cell size determinants in budding yeast.

## Highlights

• An intuitive visualization framework for cell size control is described
• Cell size control in different environments or mutant backgrounds can be compared
• Mutual dependencies between size control at different cell cycle phases are described

## Graphical Abstract

**Figure:**
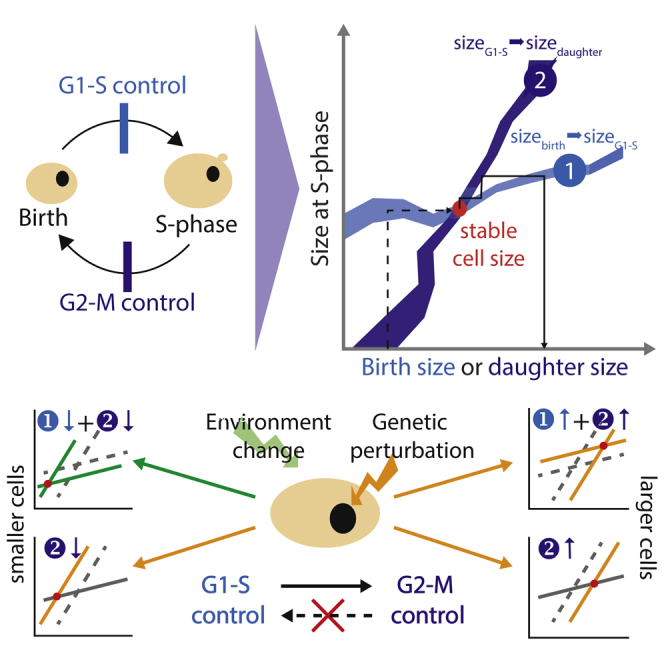

## Teaser

Jonas et al. develop a visual framework to understand the emergence of cell size from the cooperation of multiple size control mechanisms during the cell cycle. With this framework they analyze the size changes after environmental and genetic perturbations and probe the interaction between size control at different cell cycle phases.

## Introduction

Size is a defining property of cell physiology that is relatively constant within a population of identical cells but adapts readily upon changing conditions (Ginzberg et al., 2015, Rupes, 2002, Turner et al., 2012). Size control mechanisms within cells are used first to limit size fluctuations caused by stochastic variations in division time or in the rate of volume increase and second to adjust the characteristic cell size with changing conditions (Alberghina et al., 1998, Carter and Jagadish, 1978, Jorgensen et al., 2002). To achieve these functions, size control mechanisms coordinate cell cycle progression with mass accumulation (Dolznig et al., 2004, Johnston et al., 1977, Jorgensen et al., 2002). A prominent example for size control mechanisms is the size checkpoint, which halts a given cell cycle transition until a critical size threshold is reached (Fantes and Nurse, 1977, Johnston et al., 1979, Kafri et al., 2013, Nurse and Thuriaux, 1977). Studies of size control have focused on individual checkpoints that function at given cell cycle transitions, defining the molecular mechanisms regulating single transitions. Cell size, however, depends on the integrated dynamics of all these transitions, and the properties of these integrated dynamics are less well understood.

Size control mechanisms were most extensively studied in budding yeast (Carter and Sudbery, 1980, Johnston et al., 1977, Jorgensen et al., 2002). Here, size is regulated primarily at the G1/S transition, which is delayed in small-born daughter cells (Di Talia et al., 2007, Lord and Wheals, 1980). This control resembles a size checkpoint but does not impose a strict size threshold, as small-born cells still bud at a size that is smaller than the budding size of large-born cells (Di Talia et al., 2007). Consequently, the characteristic cell size depends not only on the dynamics during G1 but also on the timing and growth dynamics in the S, G2, and M phases (budded phase), which set birth size and show some properties of size control. How this interaction between both growth phases affects the characteristic cell size, however, is relatively less studied.

Recent studies provided evidence that size control is distributed throughout the cell cycle and not limited to the G1 phase alone (Anastasia et al., 2012, Chandler-Brown et al., 2017). Thus, although the volume added at G1 depends on birth size, the total volume added between two budding events is independent of the budding size, at least in diploid cells (Soifer et al., 2016). By this, budding yeast appears to comply with the “adder” mode of size control, in which cells add a constant volume between two cell cycle events. The adder model was described in different bacteria (Campos et al., 2014, Deforet et al., 2015, Sompayrac and Maaloe, 1973, Taheri-Araghi et al., 2015, Willis and Huang, 2017) and was shown to hold also in some mammalian cells (Varsano et al., 2017). Specifically in budding yeast, it is not clear whether cells effectively regulate cell cycle transitions on the basis of the added volume itself or whether an apparent adder phenomenon results from independent regulation of the G1 and budded phase (Chandler-Brown et al., 2017). Of note, the adder model does not explain how the added volume, corresponding to the characteristic cell size, is adjusted upon genetic or environmental changes.

In this study, we describe a general framework that enables intuitive visualization of the characteristic cell size, depicting this size as an emergent property of the integrated cell cycle dynamics of hundreds of individual cells. Key to this analysis is the integration of size dynamics in the different cell cycle phases. Applying this visual framework to the particular case of budding yeast, we describe how typical cell size is adjusted following a range of environmental and genetic perturbations, classifying the different perturbations on the basis of the cell cycle stage(s) that was perturbed and the means by which its growth dynamics were altered. Our results provide insights into the way cells adjust their size following perturbations and pose some challenges to existing models of cell size control, as we discuss.

## Results

### Formalism for Intuitive Visualization of the Characteristic Cell Size Emerging from the Integrated Cell Cycle Dynamics of Hundreds of Individual Cells

Eukaryotic cells increase in volume primarily in two phases: G1, which occurs after cell division and before DNA replication, and S/G2, which lasts from DNA replication to the start of mitosis. Size control can be implemented at one or both of these phases. Of note, these two phases are necessarily coupled: cell size at the end of one of these phases serves as an input to the growth control mechanism in the next phase. Accordingly, to understand how overall cell size is controlled, an integrated view of both phases is necessary.

To define a visual framework for integrated analysis of cell size control, we focused on budding yeast. Budding yeast divides asymmetrically into a large mother cell and a smaller daughter cell (Lord and Wheals, 1980). Daughter cells then enter into G1, where size control is implemented: small-born daughters extend their G1 while large-born daughters spend a shorter time in this phase. This reduces, but does not eliminate, size variations at the G1/S transition (Di Talia et al., 2007, Hartwell et al., 1973, Lord and Wheals, 1981). Cells then enter into S phase, a transition that is marked by the initiation of budding. Cells continue to grow during S/G2/M phase, with growth being directed primarily toward the bud. Following cytokinesis, the bud becomes a new daughter, initiating the next round of growth and division.

**Figure 1:**
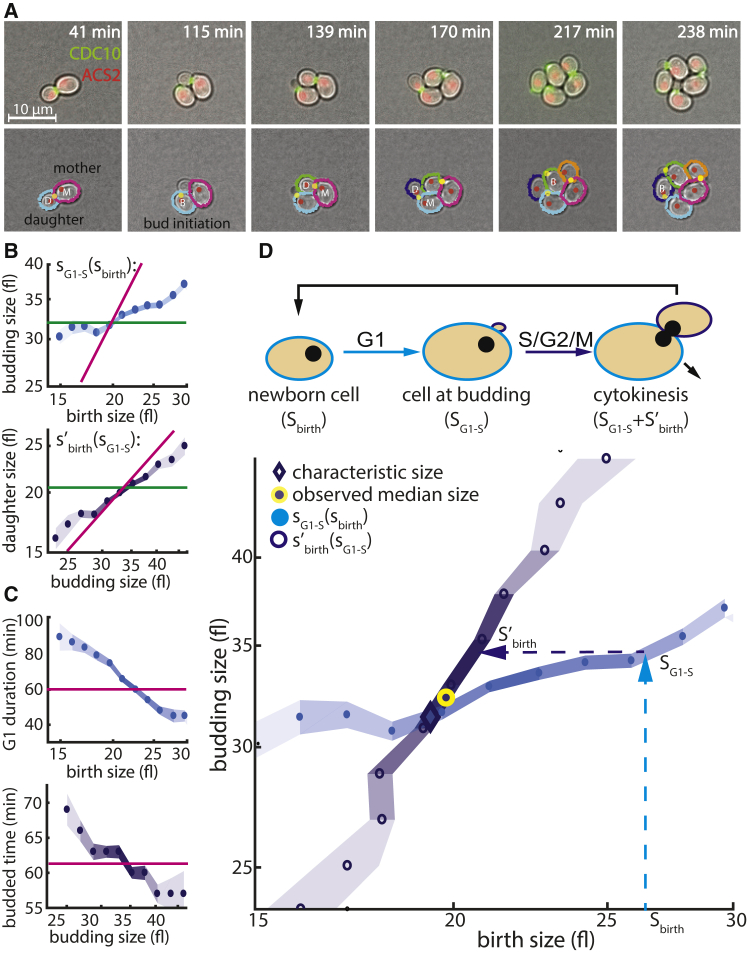
Size-Related Cell Cycle Dynamics in the Budded and Un-budded Phase (A) Live imaging and automated cell cycle analysis of a budding yeast lineage. Wild-type cells expressing two fluorescence markers, which enable the detection of key cell cycle events, CFP-tagged ACS2 (red, localized to the nucleus) and YFP-tagged CDC10 (green, localized to bud-neck), were followed using live microscopy. Shown is an exemplary time course of wild-type cells growing on glucose (top row), together with the results of an automatic image analysis used to identify cells and define their cell cycle stage on the basis of the two markers (bottom row). Here, detected cell boundaries used for size determination are shown in distinct colors, detected nuclei as red dots and detected bud necks as green dots. M,D, mother-daughter couple after cytokinesis; B, cell at budding. (B) Size mapping during the cell cycle. Top: newborn wild-type cells growing on synthetic complete (SC) medium containing 2% glucose were binned according to their birth size. Median budding size in each bin was calculated (blue circles; blue area, SE in each bin; color intensity indicates data density). Lines in this figure indicate the expected bud size in models assuming perfect size control, that is, budding size equals median budding size (green) or no size control, that is, linear relation between birth and budding size (pink; S_G1-S_/S_birth_ = 1.48). Bottom: same as above for the mapping of budding size to the next daughter size (dark blue and S′_birth_/S_G1-S_ = 0.61). (C) Size-dependent timing of cell cycle phases. Top: newborn wild-type cells growing on SC medium containing 2% glucose were binned according to their birth size, and the median un-budded phase (G1) duration in each bin was calculated (blue circles; blue area, SE in each bin; color intensity indicates data density; pink line indicates median G1 duration that would be expected without size control). Bottom: median budded phase duration as a function of budding size (dark blue). (D) Median cell size defined by the size-related cell cycle dynamics. Top: schematic description of relation between the two size mapping functions, S_birth_ → S_G1-S_ (blue) and S_G1-S_ → S′_birth_ (dark blue) in a growing population. Bottom: the two size-mapping functions from (B) are plotted on the same axes. Predicted median birth and budding sizes are defined by the intersection of these two mapping functions (dark blue diamond), and the observed median is highlighted in yellow.

To understand how the typical cell size emerges from these integrated dynamics, we have followed a lineage of cells across multiple generations, focusing, at each generation on only the first cell cycle of new-born daughter cells (Figures 1A and 1D). A cell that is born at some size, S_birth_, will grow during G1 and initiate DNA replication and budding at some larger size, S_G1-S_. The bud will then grow and separate from its mother to initiate the next daughter cell of a size S′_birth_. For a population to maintain a constant characteristic size, these dynamics should converge to a steady state at which the median daughter size, S′_birth_, is the same as the median birth size in the current cycle, S_birth_. This steady state then defines the typical cell size of the respective growing population.

To examine experimentally the size-mapping dynamics between sizes at the G1/S transition and at birth, we used the setup described by Soifer and Barkai (2014) to follow ∼1,000 individual new-born cells. For every cell, we determined the size at birth (S_birth_), size at the start of S phase, that is, budding (S_G1-S_), and the birth size of its first daughter (S′_birth_) (Figure 1A, bottom). The relations among these three parameters define the size-related mapping from birth to budding and from budding to the birth of its first daughter (Figure 1B). Because cells grow exponentially, we consider here and below the logarithmic of size, s = log(S). To visualize the extent of size control, we also plotted two additional lines, simulating the expected mapping in the absence of size control (pink line, slope = 1) and in the presence of a perfect, checkpoint control (a constant transition size independent of the size at the onset of the respective phase; Figure 1B, green line).

Consistent with previous results, the median budding size, s_G1-S_, shows only a minor dependency on birth size, s_birth_, indicating a prominent size control in G1 (i.e., slope < 0.5; Figure 1B, top). Notably, as expected, sizes s_G1-S_ (and s′_birth_) were symmetrically distributed around a median value (Figure S1). Size regulation was observed also in S-G2 but was less prominent, with s′_birth_(s_G1-S_) being closer to the linear dependency expected in the absence of size control (i.e., slope > 0.5; Figure 1B, bottom). This is further evident when examining the duration of both phases; although a clear signature of size control was seen in both phases, as G1 duration decreases with birth size and, similarly, budded phase duration decreases with budding size (Figure 1C), this dependency was stronger in G1 than in the budded phase. This is consistent with previous results showing that size regulation in the budded phase becomes prominent only in small-budded cells (Soifer and Barkai, 2014).

To examine how the typical cell size emerges from the combined size-dependent dynamics, we compare the two size-mapping relations on the same plot (Figure 1D). In this form, the x axis represents either the birth size of a given cell (used in size mapping from birth to budding, i.e., s_birth_ → s_G1-S_) or the birth size of its first daughter (used in the second size mapping from budding to first daughter, s_G1-S_ → s′_birth_). The intersection of these two monotonic curves represents the only stable steady state at which the median sizes are maintained between generations. This intersection thereby corresponds to the average birth and budding size of a stably growing population. Indeed, the observed median birth and budding size of wild-type cells largely reproduced this theoretical value (Figure 1D).

We next apply this formalism to examine how the characteristic cell size is adjusted by different environmental or genetic perturbations.

### The Contributions of G1 and Budded Phases to Nutrient-Dependent Size Adjustment

**Figure 2:**
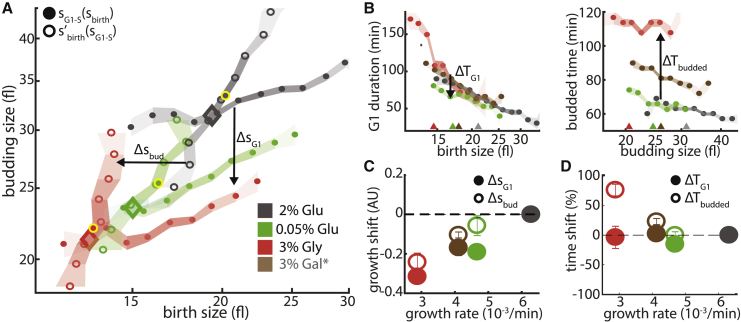
Slowing Growth by Changing Carbon Source Decreased Growth in Both G1 and the Budded Phase (A) Size mapping during the cell cycle. Same as Figure 1D for cells growing on indicated carbon sources (arrows indicate size mapping change, i.e., growth shift, in G1 [Δs_G1_] and the budded phase [Δs_bud_]) (here and following figures: gray, 2% glucose; green, 0.05% glucose; red, 3% glycerol; size mapping of wild-type cells in galactose [brown] can be found in Figure S2). (B) Size-dependent cell cycle timing, Same as Figure 1C for cells growing on different carbon sources (annotated as in A; arrows indicate time shift in G1 [ΔT_G1_] and the budded phase [ΔT_budded_]; colored triangles indicate median birth and budding size; colored areas indicate SE; color intensity indicates data density). (C) Size dynamics correlate with growth rate. Shown are the growth shifts calculated in log-space for cells growing on different carbon sources against growth on 2% glucose; for example, a growth shift of 0.1 AU corresponds to e^0.1^ ∼ 10% bigger budding size for the same birth size (Δs_G1_ and Δs_bud_; see A) as a function of the cells’ specific growth rates (annotated as in A; error bars indicate SE). (D) G1 timing is not affected by carbon source, but budded phase timing is. Shown is the shift in the relative size-related phase duration for cells growing on different carbon sources against 2% glucose (ΔT_G1_/T_G1_ and ΔT_budded_/T_budded_) (see B) as a function of the cells’ specific growth rates (annotated as in A; error bars indicate SE).

Low-quality nutrients decrease the specific growth rate, μ, of cells and correspondingly increase their division time (T_d_ = ln[2]/μ). Typically, these cells are smaller (Johnston et al., 1979, Tyson et al., 1979). We used our framework to examine how the characteristic cell size is adjusted with the available nutrient. To this end, we compared cells growing on different carbon sources corresponding to a progressively slower growth rate: high glucose concentration, low glucose concentration, galactose, or glycerol. For each medium, the cell cycle dynamics of several hundred individual cells were followed (Figure 2).

During growth in poor nutrients, both size-control maps, describing the dynamics in the un-budded (G1) and budded phases. shifted to smaller sizes, that is, down and left (Figures 2A and S2). Consequently, the characteristic size, defined by the intersection of these curves, also decreased and well captures the observed median size in each medium.

Slower growth rate necessarily implies a proportionally longer cell cycle. Notably, and as we showed before (Soifer and Barkai, 2014), the increased G1 duration of the slow-growing cells was fully explained by their reduced birth size, so that cells that were born at a given size remained in G1 for the same duration, independently of the available nutrient (Figure 2B, left). Slow-growing cells still accumulate less volume during this time, explaining the observed shift in the size-control map (Figures 2C and 2D, filled circles). In contrast, the duration of the budded phase was greatly extended in poor nutrients even when comparing cells that budded at a similar size (Figure 2B, right), but this increase in time (i.e., time shift) was not sufficient to compensate for the reduced specific growth rate, leading to reduced volume accumulation also in this phase (Figures 2C and 2D, empty circles).

### Slowing Growth by Forcing Excess Protein Expression Increases Cell Size through a Coordinate Shift of the Size-Control Mapping in Both the Budded and Un-budded Phases

Recently, we and others have noted that slowing cell growth by forcing expression of excess protein or mRNA reduces the specific growth rate but increases cell size (Basan et al., 2015, Kafri et al., 2016a). These so-called burden cells showed a size increase that was proportional to the introduced burden and extended the duration of the cell cycle. To understand how they adjusted their characteristic size, we followed >1,000 individual burden cells with extensive mRNA or protein expression.

**Figure 3:**
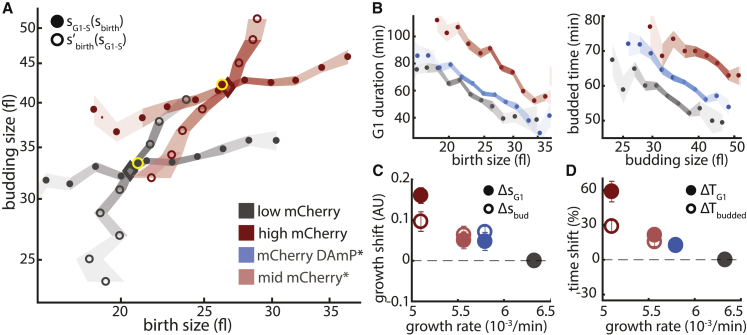
Slowing Growth by Forcing Excess Protein Production Coordinately Shifts Growth up in Both G1 and the Budded Phase (A) Size mapping during the cell cycle. Cells were forced to express high amounts of mCherry proteins using stable or unstable mRNA. Shown is the integrated size mapping, s_birth_ → s_G1-S_ and s_G1-S_ → s′_birth_, for low and high translation burden (here and following figures: gray, low mCherry expression; light red, medium mCherry expression [size mapping in Figure S3B]; dark red, high mCherry expression; blue, high expression of unstable DAmP-mCherry mRNA [Schuldiner et al., 2005]; size mapping in Figure S3B). (B) Size-dependent cell cycle timing. Same as Figure 2B for the indicated burden cells. (C) Size dynamics correlate with growth rate. Same as Figure 2C for the indicated burden cells. (D) Time shifts correlate with growth rate. Same as Figure 2D for the indicated burden cells.

Analyzing these data using our formalism revealed that both protein and mRNA burden causes cells to shift their size control maps toward larger sizes, leading to a larger characteristic size (Figures 3A and S3B). Notably, the extent of the upshift in cells with solely increased mRNA transcription from multiple mCherry-DAmP (Schuldiner et al., 2005) copies was similar to the changes in protein-expressing cells with the same growth defect, suggesting that increased size is independent of protein accumulation and directly results from the reduced growth rate (Figures S3B–S3D). Furthermore, this size increase was also observed when growing burden cells in media containing low glucose concentrations (Figure S3A).

An upshift was also observed when examining the durations of the two cell cycle phases: burdened cells extended G1 even relative to wild-type cells that were born at the same size (Figure 3B). Comparing sub-optimal growth conditions and forced protein expression suggests that slowing growth can lead to either up- or downshifts in the size control mapping, depending on the perturbation types (Figures 2 and 3).

### Classifying Mutants on the Basis of Their Effects on the Size-Control Maps

Our analysis above suggested that slow growth can affect cell size in different ways, depending on the perturbation type. However, both perturbation types we examined above, sub-optimal carbon sources and protein burden, shifted the two size-control maps in a coordinated manner and in proportion to the change in cell growth rate (Figures 2C, 2D, 3C, and 3D). To better understand whether this coordination is observed more generally, and to gain insights into the genetic basis of the shifts, we examined gene deletions that perturb cell size and growth rate (Dungrawala et al., 2012, Ohya et al., 2005, Zhang et al., 2002). To this end, we re-visited data we have previously reported, describing the cell cycle dynamics of several hundred size-perturbed mutants (Soifer and Barkai, 2014), and analyzed it using our framework.

**Figure 4:**
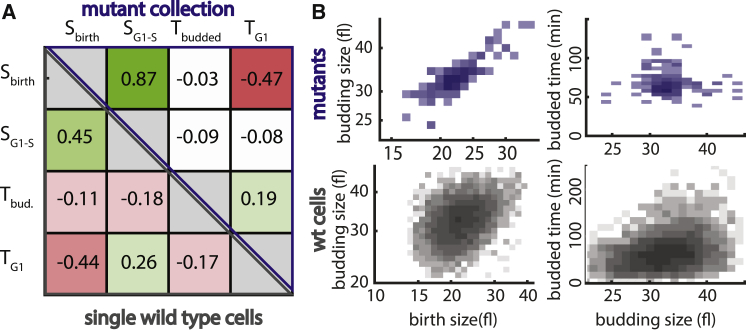
Integrative Analysis of Size-Perturbing Gene Deletions (A) Median values of growth parameters in mutant strains showed a similar correlation pattern to that observed at the single-cell level. Correlations between birth size (S_birth_), budding size (S_G1-S_), G1 duration (T_G1_), and budded phase duration (T_bud._, T_budded_) are shown (upper right triangle, correlation between median values found in the size-perturbed deletion strains [see Soifer and Barkai, 2014, for details and the Supplemental Information for a list of mutants]; lower left triangle, correlation between >1,000 individual wild-type cells). (B) Birth and budding size positively correlate in individual cells and across mutant strains. Median budding size is plotted with median birth size (left) or budded time (right) for all mutant strains (blue, top) or individual wild-type cells (gray, bottom) between the indicated parameters as density maps (size axes in log scale).

For each mutant, microscopic analysis of cell cycle dynamics of hundreds of individual cells defined cell size at budding and at birth, as well as the durations of G1 and budded phase. When compared between mutants, median values of these parameters showed a correlation pattern that was similar to that observed at the level of individual cells (Figures 4A and 4B): birth size was positively correlated with budding size but negatively correlated with G1 duration. Correlations were significantly weaker when considering the budded phase.

**Figure 5:**
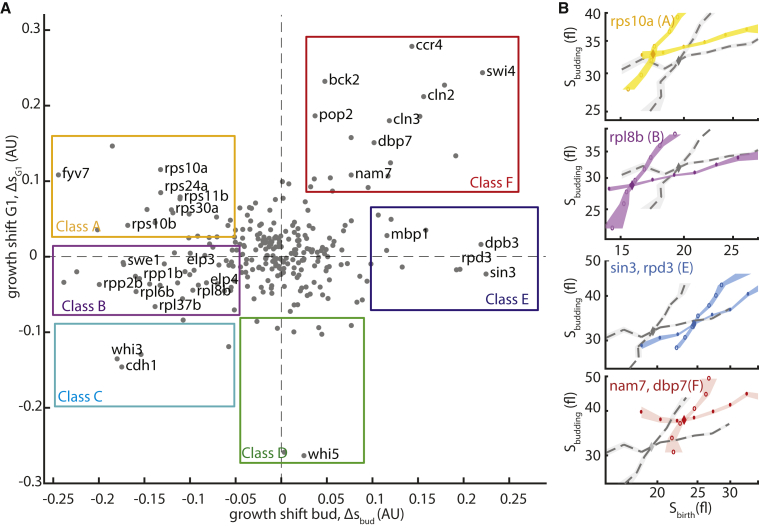
Shifts in Size Mapping after Gene Deletion (A) Mutant classification on the basis of size mapping change in both cell cycle phases. Shown are the calculated growth shifts in G1 (Δs_G1_) and the budded phase (Δs_bud_, calculated as in Figures 2C and 3C and illustrated in Figure 2A) for all mutants in the dataset with n > 100 newborn cells (colored rectangles, mutant classification). (B) Size mapping of major mutant classes. The size mapping of exemplary mutants for each class is shown (color and letter in parenthesis: mutant class; from top to bottom: class A, rps10a and rps30a; class B, rpl8; class E, sin3, rpd3; class F, dbp7, nam7).

Applying our formalism, we determined the shifts in the size-controls maps (i.e., growth shift) in both the G1 and budded phase, as above (Figure 5A). This time, we observed a range of different and partially decoupled behaviors. On the basis of these shifts, we classified the mutants into six subtypes, as shown. Mutants in classes A–D were smaller than the wild-type, while these in classes E and F were larger (see Table S1 for median sizes).

### Differential Effects of Protein Translation and mRNA Processing on Size Regulation

Most mutants in our dataset were assigned to classes A and B (small mutants) and classes E and F (large mutants). Classes A and B included mostly mutants associated with translation and were correspondingly slow growing. Both classes shifted the size-control map in the budded phase toward lower sizes but were distinguished on the basis of their G1 dynamics (Figure 5B, top rows): mutants in class B, which includes most large ribosomal subunit deletions, showed a minor size-decreasing shift also in G1, while mutants in class A, which includes most small ribosomal subunits deletions, showed a major size-increasing shift that was proportional to the change in growth rate (Figure S5). These differential effects concurred with the changes in G1 timing: size-dependent G1 duration increased in deletions in the small ribosomal subunit but remained invariant to deletions in the large ribosomal subunit, as reported previously (Soifer and Barkai, 2014).

Mutations that increased cell size were also separated into two classes (E and F). Mutants in both classes shifted the size control maps in the budded phase toward larger sizes but were, again, distinguished on the basis of their G1 dynamics (Figure 5B, bottom rows): mutants in class E, such as SIN3 or RPD3 deletion, maintained G1 growth largely unchanged, while class F mutants showed a size-increasing shift also in G1 that was quantitatively correlated with the shift observed in the budded phase. This later phenotype, seen in class F mutants, largely resembled that of the burdened cells. Included in class F were the majority of G1-effectors genes, as well as slow-growing mutants associated with the CCR4-NOT complex (e.g., ccr4, pop2), which is involved in several key mRNA metabolic process (Tucker et al., 2001). This large-cell phenotype of CCR4-NOT perturbation was reported before (Manukyan et al., 2008). Thus, although deletion of CCR4-NOT subunits decreased growth rate by globally perturbing the process of protein production, similarly to mutations in ribosomal proteins, they showed an opposite effect on cells size.

### G1/S Effectors’ Impacts on Volume Changes during Both G1 and the Budded Phase

**Figure 6:**
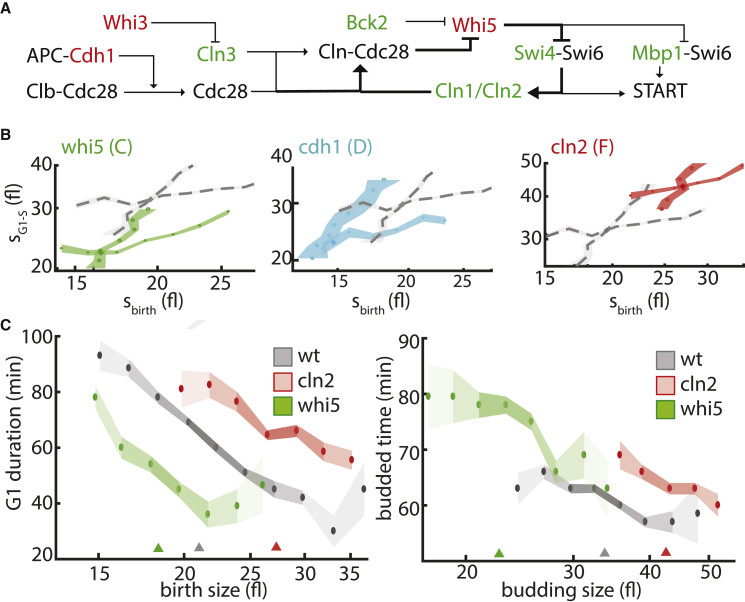
Mutants Involved in the G1/S Transition Modulate Growth in Both G1 and the Budded Phase (A) Genetic network underlying START transition. The diagram shows the activators (green) and inhibitors (red) of G1-to-S progression and their interactions (arrow, activating; bar, inhibiting interaction) in *S. cerevisiae* (thick lines, positive feedback [FB] loop enabling switch-like behavior). (B) Size mapping after cell cycle perturbations. Exemplary size mappings and classes of cell cycle mutants (color and letter in parenthesis: mutant class; from left to right: whi5, class C; cdh1, class D; cln2, class F). (C) Size-dependent cell cycle timing. Same as Figure 2B for the indicated strains (colored triangles, median birth and budding size of each mutant).

Next, we examined more closely how size is adjusted following deletion of known cell cycle regulators (Figure 6A). Among the smallest mutants in our dataset were whi5 and swe1, deletions of two known cell cycle regulators that respectively function during G1 and the budded phase (Booher et al., 1993, Costanzo et al., 2004, de Bruin et al., 2004, Kellogg, 2003). WHI5, the principle inhibitor of the G1/S transition, was assigned to class D mutants (Figure 6B, left): shifting the size control downward in G1 but showing no effect on the budded phase. Similarly, SWE1 deletion (class B) specifically affected the budded phase, but showed a limited effect on G1 (Figure S4A).

In contrast to the phase-specific phenotype of WHI5 and SWE1, most other START regulators affected both phases (Figure 6B). Thus, deletion of *WHI3*, a negative regulator of the WHI5 antagonist CLN3 (Garí et al., 2001, Nash et al., 2001), or of the G1-specific APC activator CDH1 (Schwab et al., 1997, Visintin et al., 1997), shifted the size-control maps downward in both G1 and the budded phase (Figure 6B, middle). Similarly, deletion of positive regulators of the G1/S transition, such as CLN2, SWI4, or CLN3 (Cross, 1988, de Bruin et al., 2004, Hadwiger et al., 1989), led to the opposite shift (class F; Figure 6B, right). BCK2 (Di Como et al., 1995) was an exception, showing a rather specific effect on the G1 phase (Figure S4B).

Of note, the specific growth rates of these cell cycle mutants remained largely intact despite the shift in the size mapping in both G1 and the budded phase. This was explained when examining the duration of these phases: both size-mapping curves were shifted, but these shifts were alleviated by change of the median sizes at birth and budding (Figure 6C).

### Budded Phase Size-Related Dynamics Are Independent of whi5 or G1 Growth

The finding that most START regulators affect not only the G1 phase but also the budded phase was unexpected. As we described above, these effects were similar to these observed when forcing excess of protein expression and upon deletion of CCR4-NOT components. In all these cases, the shifts in the size-control mappings were correlated between the two phases. We therefore asked whether perturbations propagate from G1 phase to the budded phase, or inversely, whether perturbations that affect the budded phase propagate to affect the G1 phase.

**Figure 7:**
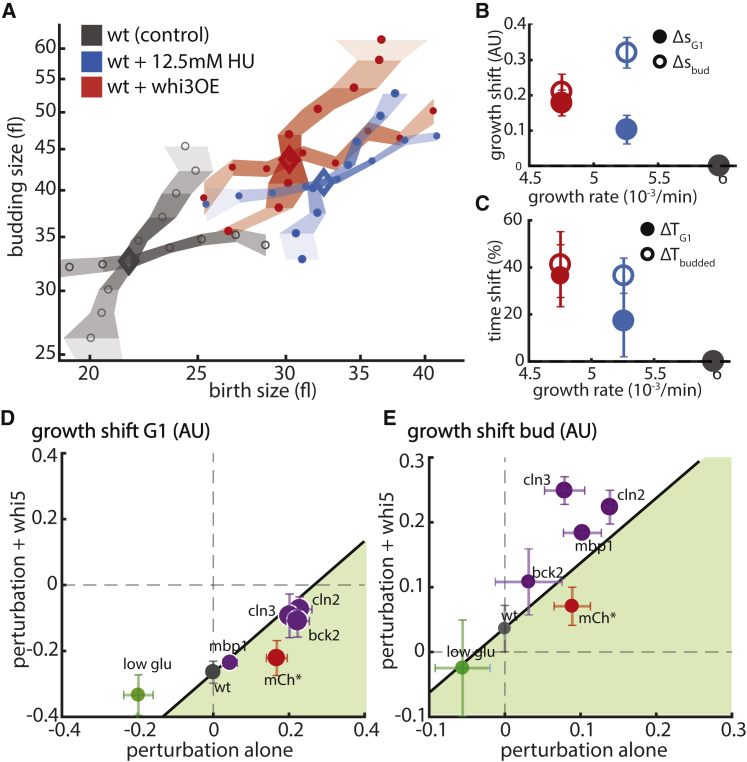
Growth Change Propagates Stronger from G1 to the Budded Phase Than Vice Versa (A) Size mapping of wild-type cells after phase specific perturbations. Size mapping of wild-type cells after whi3 overexpression (repressor of START) or in 12.5 mM HU (slowed S phase) is shown as in Figure 2A (gray, unperturbed wild-type cells; blue, 12.5 mM HU; red, whi3 overexpression). (B and C) Growth (B) and time (C) shift versus growth rate after phase-specific perturbations. Same as Figures 2B and 2C for indicated perturbations. (D and E) WHI5 is not required for growth propagation from G1 to budded phase. Size-changing perturbations were performed in wild-type and whi5-deficient cells, and the plots show the growth shift (calculated against unperturbed wild-type cells) in WHI5-deficient cells (y axis) and wild-type cells (x axis) in G1 (D) or the budded phase (E) (gray dot, growth shift after WHI5 deletion; purple dots, additional deletion of cell cycle regulators MBP1, CLN2, CLN3, and BCK2; red dot, mCherry overexpression; green dot, growth in 0.05% glucose; black line, growth effect that can be expected from a purely additive effect; green area, less additional growth after WHI5 deletion).

To examine that, we performed two additional experiments (Figure 7A). First, we overexpressed *WHI3*, an inhibitor of CLN3. Similar to all other START effectors, also this overexpression led to an upshift of both size-control curves (Figures 7A and 7B). Second, we subjected cells to low levels of hydroxyurea, which specifically delays S-phase progression via dNTP depletion (Figure 7C). This addition had small effect on growth rate but caused a significant upshift of budded phase size-control mapping, as expected (Figures 7A and 7B). Of note, in this case, the G1 size-control map was hardly affected. Together, these results suggest that although the budded phase can be perturbed with little consequences on G1, perturbations affecting the G1 size control propagate to influence the budded-phase dynamics.

WHI5 deletion was an exception, as deletion of the START regulator specifically perturbed G1 dynamics, with no consequences on the budded phase. We therefore examined whether deletion of WHI5 also eliminates the effects of the other START mutants on budded-phase dynamics. To this end, we deleted WHI*5* in cells deleted of CLN2, CLN3, and MBP1 as well as in the burden strains forced to express high mCherry levels (Figures 7D and 7E). In all cases, deletion of WHI5 shifted the G1 control curves toward smaller size (Figure 7D) but had little impact on the budded phase (Figure 7E), as expected in the case of additive effects (Figures 7D and 7E, black line). Only for the burden strain did we observe a small signal suggesting the possibility of an epistatic interaction (Figures 7D and 7E, green area). Together, these results suggest that the propagation of effects from START effectors to the budded phase is independent of WHI5.

## Discussion

Size control mechanisms link cell cycle progression to cell size (Johnston et al., 1977, Jorgensen et al., 2002). In most cells, this link is commonly established at the transition from a growth phase (G1 or S/G2) to the next step in the cell cycle. Budding yeast, for example, minimizes size fluctuations through a size-dependent gating at the G1/S transition, but other organisms use a G2/M checkpoint to achieve size control (Nurse, 1975). Extensive studies, mostly in budding yeast, characterized the molecular mechanisms that function at those control points (Cross, 1988, Di Talia et al., 2007, Jorgensen et al., 2002, Polymenis and Schmidt, 1997, Skotheim et al., 2008). Here, we focus our analysis on the question of how the integrated growth dynamics over the whole cell cycle shape the characteristic cell size and how cells adjust their size following a range of perturbations. To this end, we present an intuitive visualization scheme that can be applied in a wide range of cell types. Specifically, by simultaneously plotting the growth dynamics in both growth phases, we can appreciate the strength of size control at each individual phase and understand how the integrated function of both control mechanisms determines the cell size. This visualization depends on single-cell data that can be obtained for every cell type for which visual cell cycle markers are available. This includes the fluorescence ubiquitination cell cycle indicator (FUCCI) system in mammalian cells (Sakaue-Sawano et al., 2008) or bud neck appearance in *S. cerevisiae*.

We have applied this framework for analyzing cell-size properties of budding yeast. Similarly to other microbes, budding yeast growing in less preferred media decreases its size in proportion to the change in growth rate (Jagadish and Carter, 1977, Tyson et al., 1979). Using our framework, we show that this size adjustment depends not only on changes in the size-gating properties at the G1/S transition but also on a pronounced adjustment of budded-phase dynamics. More specifically, the size-control mappings were shifted toward smaller sizes both in G1 and in the budded phase.

Notably, the observed downward shift in the size-control mapping of the budded phase during growth in low-carbon was recapitulated in mutants deleted of ribosomal subunits. This may suggest that absolute growth during this phase scales with global translation capacity. As ribosome content of cells growing on different carbon sources scales with growth rate (Metzl-Raz et al., 2017), this could explain the change in the budded phase size-control mapping. Of note, in contrast to their consistent effect on the budded-phase dynamics, ribosome mutants showed differential effects on the size-control mapping in G1, as this mapping was shifted downward upon deletion of large ribosomal subunits but upward when deleting small ribosomal subunits. This may indicate a more direct role of translation initiation in sensing cell size at the G1/S transition, as previously suggested (Barbet et al., 1996, Brenner et al., 1988, Hanic-Joyce et al., 1987, Polymenis and Schmidt, 1997, Soifer and Barkai, 2014). Mechanistically, this could be implemented if perturbed translation initiation hinders the production and accumulation of key G1/S regulators (e.g., CLN3, SWI4), which have exceptionally long 5′ UTRs and whose concentration is critical for G1-S transition (Dorsey et al., 2018).

Contrasting the tight correlation between cell size and cell growth rate in different nutrients, slowing growth by forced protein or mRNA expression increased, rather than decreased, cell size (Kafri et al., 2016a). Excess protein translation is therefore unlikely to explain the slow growth or increased size of burdened cells. Rather, the large-size phenotype of the burden cells was mimicked by mutations in the CCR4-NOT mRNA processing complex, suggesting that also mRNA-related processes become limiting in those cells and further that these processes affect the size control in both G1 and the budded phase (Kafri et al., 2016a, Kafri et al., 2016b).

Coordinated shifts in the size dynamics of both G1 and the budded phase were observed not only in the burden cells and CCR4-NOT mutants but also upon deletion of G1/S regulators, including CLN2, SWI4, and CLN3, or after WHI3 overexpression. One possible explanation for this coordination is that the duration of the budded phase is communicated to delay START, for example, by increasing production of START inhibitors, such as WHI5 which accumulate during this phase (Schmoller et al., 2015). However, this appears unlikely because specifically increasing budded-phase duration by the addition of HU or in mutants such as RPD3 deletion had no significant effect on G1 size control. Rather, it appears that perturbations that promote or delay the G1/S transition lead to a similar effect on the budded phase dynamics. Further studies should address how this propagation occurs at the molecular level and why WHI5 deletion does not show such an effect.

## STAR★Methods

### Key Resources Table

**Table:** 

REAGENT or RESOURCE	SOURCE	IDENTIFIER
**Chemicals, Peptides, and Recombinant Proteins**		

Hydroxyurea (HU)	Bio Basic	Cat#HB0528
SeaPlaque TM Agarose	Lonza	Cat#50100

**Critical Commercial Assays**		

HiYield Plasmid Mini Kit	RBC Bioscience	Cat#YPD100

**Deposited Data**		

Cell cycle files from processed videos	This study	https://doi.org/10.17632/t4n9mys2sm.1
Cell cycle files from processed videos	Soifer and Barkai, 2014	https://doi.org/10.17632/t4n9mys2sm.1

**Experimental Models: Organisms/Strains**		

YFJ100 (BY4741 MATa, his3-1, leu2-0, met15-0, ura3-0, ACE2::Ace2p-CFP KanMX, CDC10:Cdc10p-YFP NatA)	This study	N/A
YFJ101 (BY4741 MATa, his3-1, leu2-0, met15-0, ura3-0, ACE2::Ace2p-CFP KanMX, CDC10:Cdc10p-YFP NatA, WHI5::URA3)	This study	N/A

**Recombinant DNA**		

pBS35TDH3	Kafri et al., 2016a	N/A
pBS69TDH3	Kafri et al., 2016a	N/A
pBS35WHI3	This study	N/A
pKT103NatA	Sharon et al., 2012	N/A
pYM30	Janke et al., 2004	N/A

**Software and Algorithms**		

MATLAB	Mathworks	N/A
Image processing algorithm	Soifer and Barkai, 2014	https://doi.org/10.17632/t4n9mys2sm.1

### Methods

#### Contact for Reagent and Resource Sharing

Further information and requests for resources and reagents should be directed to and will be fulfilled by the Lead Contact, Naama Barkai (naama.barkai@weizmann.ac.il).

### Experimental Model and Subject Details

#### Yeast strains and genetic manipulations

The construction and imaging of most mutant strains was already described previously (Soifer and Barkai, 2014). For transcriptional and translational burden, a wild-type-like strain with cell cycle markers (CDC10-YFP and ACS2-CFP) was created by genomic integration of a modified pKT103 plasmid (Sharon et al., 2012) and pYM30 (Janke et al., 2004) into BY4741 using the NatA and G418 resistance selection, respectively. This strain was subsequently used to create the WHI5 deletion strain by integrating the URA marker into the WHI5 locus. Subsequently, translational and transcriptional burden strains were constructed by multiple insertion of pBS35TDH3 (damp mCherry under TDH3 promoter without terminator) or a modified pBS34 with hygromycin resistance (pBS69TDH3, mCherry under TDH3 promoter with terminator) into the TDH3 locus. mCherry copy number and growth rate defect of burden strains were determined with flow cytometry, quantitative real-time PCR or competition experiments, as described previously (Kafri et al., 2016a). The WHI3 overexpressing yeast strain was created by using the same technique to integrate multiple copies of the WHI3 gene and hygromycin resistance into the WHI3 locus.

### Method Details

#### Time-lapse microscopy of burden strains

Cells were pre-grown for around 12 hr in SC medium to OD600 of about 0.2 in either 2% or 0.05% glucose. The cells were then prepared for imaging on agar pads in 96-well plate with the respective medium as previously described (Bean et al., 2006). Growth of micro colonies at 30°C was observed with a fully automated Zeiss Axio Observer Z1 inverted microscope equipped with a motorized XY and Z stage, external excitation and emission filter wheels (Prior), IR-based Definite Autofocus from Zeiss and a 63 × oil objective. Fluorescent proteins were detected with the 46 and 47 filter set from Zeiss for YFP and CFP respectively. Exposure times and fluorescent intensity for the YFP or CFP detection were 150 and 100 ms. Images were acquired with the Orca FLASH 4.0 v2 CMOS camera (Hamamatsu). The microscopic setup allowed sequential imaging of bright field (- 1.5 um offset for image analysis), and both fluorescence channels for 20 positions in 3 min and over 6 hr.

### Quantification and Statistical Analyses

#### Image processing

Identification and tracking of dividing cells was performed by custom-written software in MATLAB (Mathworks). Movies were analyzed from the end to the beginning, first segmenting cells based on their high contrast outline in the last image and then tracking them to the first image. Additionally, the nuclear marker facilitated the initial tracking and segmentation. Nuclear separation was identified by appearance of the nuclear marker in the daughter cell. Cell birth, defined by the bud neck disappearance, was identified as a significant decrease in the intensity of the bud neck marker in proximity (up to 30 min) to the nuclear separation. Cell volume was estimated from the bright field images assuming that the yeast cells are prolate spheroids (Lord and Wheals, 1981). The results remain qualitatively the same when considering the area of the cell instead of the volume (see Soifer and Barkai, 2014 for details).

### Data analysis

To determine the relative sizing change of each mutant or the burden strain to the wild-type, we first binned the cells according to their birth or budding size (∼10% variation between the biggest and the smallest cell in each bin) and calculated the median budding or daughter birth size in each bin with more than 5 cells. The weighted mean difference between the budding or daughter birth size (in log-space) between selected strain / conditions and the wild-type over all overlapping bins was subsequently calculated as delta growth in the G1 and budded phase, respectively. Its standard error was subsequently determined, as the weighted standard deviation between the mean differences in all samples. To prevent artifacts stemming from the finite lengths of our movies, we considered only cells born at least 100 min prior to the end of the move in our analyses. (Data for the mutant strains was previously collected for Soifer and Barkai, 2014). The time shift was analogously calculated by taking the weighted mean of the differences between the median G1 / budded phase duration in the different birth / budding in each bins and dividing it by the median G1 / budded of wild-type cells in 2% glucose.

### Data and Software availability

The files after image processing and the image processing pipeline are available on Mendeley Data (https://doi.org/10.17632/t4n9mys2sm.1).

## Supplemental Information

Document S1. Figures S1–S5

Table S1. Median Growth Parameters of Investigated Mutant Strains, Related to Figure 4 Standard name, systematic name, median birth, and bud size as well as the growth and time shifts of each investigated mutant.

Document S2. Article plus Supplemental Information
