# Supplementary material for: A Visual Framework for Classifying Determinants of Cell Size
Source: Cell Rep. 2018 Dec 18;25(12):3519–3529.e2. doi: 10.1016/j.celrep.2018.11.087 (PMC6315284; doi:10.1016/j.celrep.2018.11.087)
Supplement: Document S1. Figures S1–S5 [file mmc1.pdf]

Cell Reports, Volume *25*

Supplemental Information

A Visual Framework for Classifying Determinants of Cell Size

Felix Jonas, Ilya Soifer, and Naama Barkai

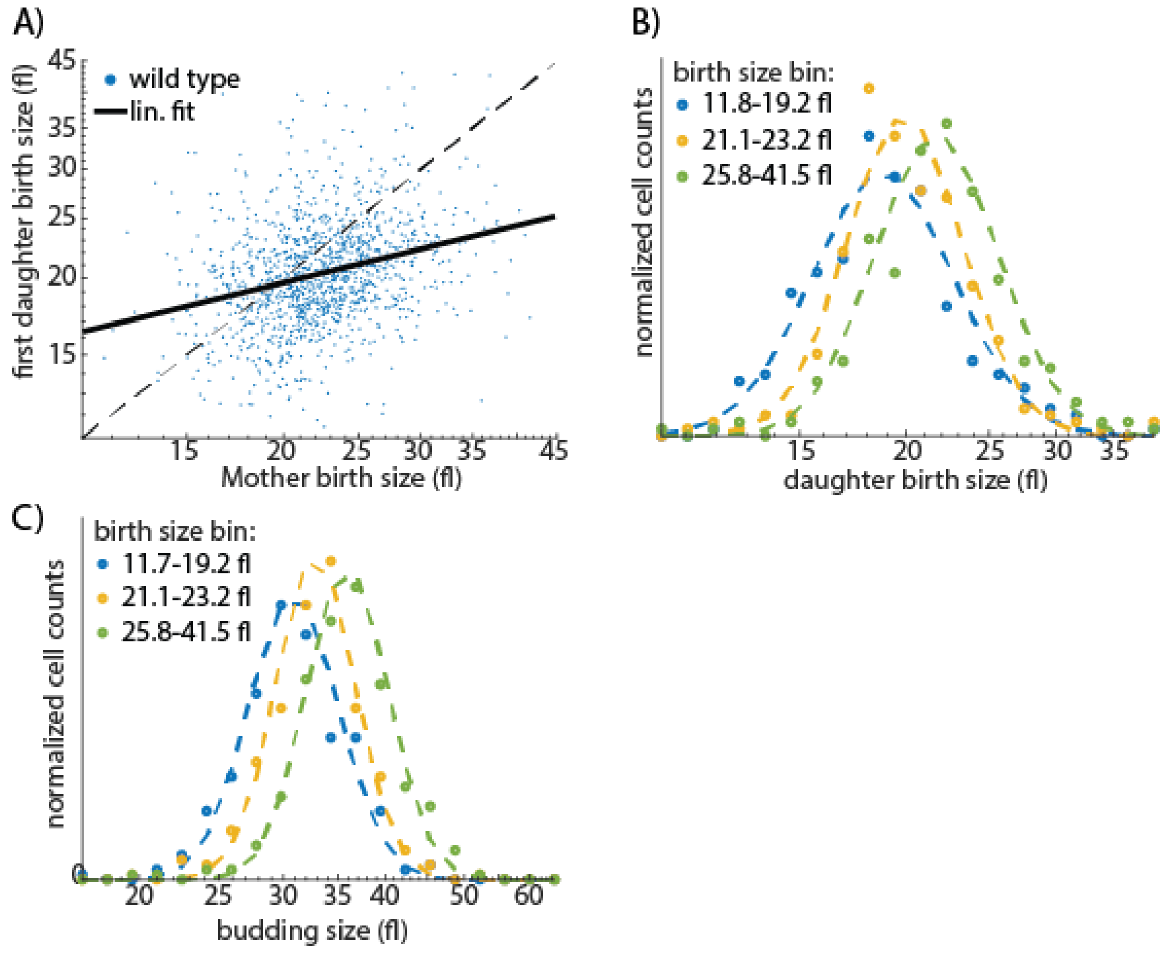

Figure S1: Daughter and budding size ($s_{G1\text{-}S}$) distribution for different birth sizes, related to Figure 1: A) *Size dynamics over a full cell cycle:* Mother birth and first daughter birth size for all recorded cell wild type cycles (blue dots, black solid line: linear fit, dashed line: no size control). B) First daughter size distributions for three different birth size bins (blue: small, yellow: normal, green: big, dashed lines correspond to fitted normal distributions) of wild type cells on 2% glucose. C) Budding size distributions for the birth size bins in (B).

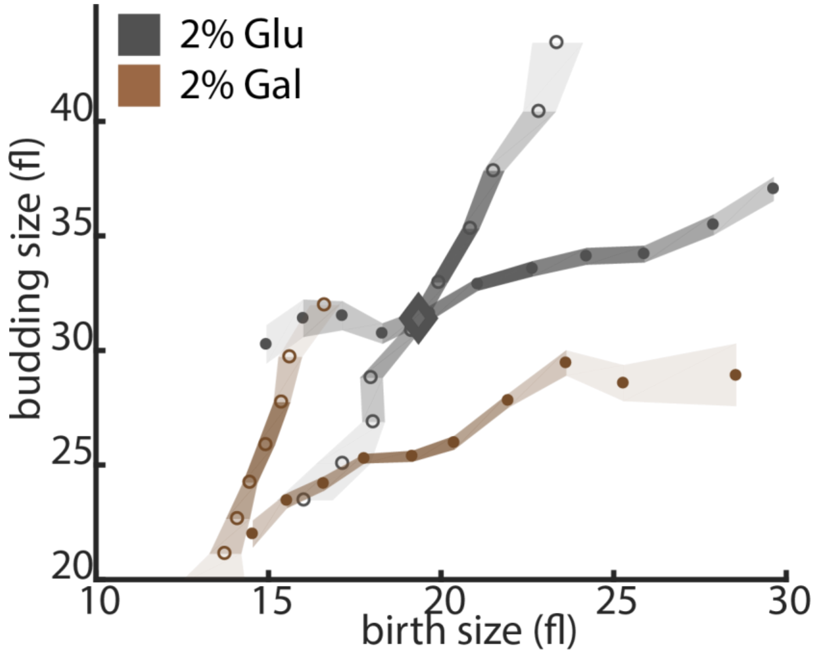

Figure S2: Slowing growth by changing carbon source decreased growth in both G1 and the budded phase, related to Figure 2: *Size mapping during the cell cycle:* Same as Fig. 2A for cells growing on the 2% galactose (grey: 2% glucose; brown: 2% galactose).

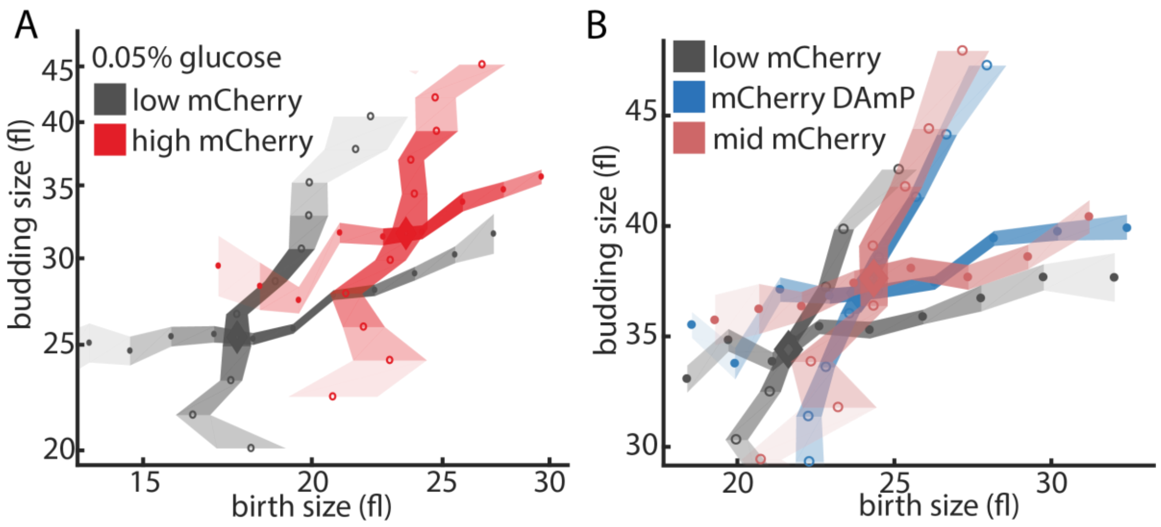

Figure S3: Burden effect is independent of nutrient condition or protein translation, related to Figure 3:

A) *Size mapping of burden cells in low glucose:* Same as Fig. 3A for burden cells growing on 0.05% glucose (grey: low mCherry expression; red: high mCherry expression).

B) *Comparative size mapping of transcriptional and translational burden cells:* Same as Fig. 3A for wild type cells expressing high levels of destabilized DamP-mCherry transcripts or normal mCherry transcripts with a similiar growth defect (grey: low mCherry expression; blue: high DamP mCherry expression; light red: medium mCherry expression)

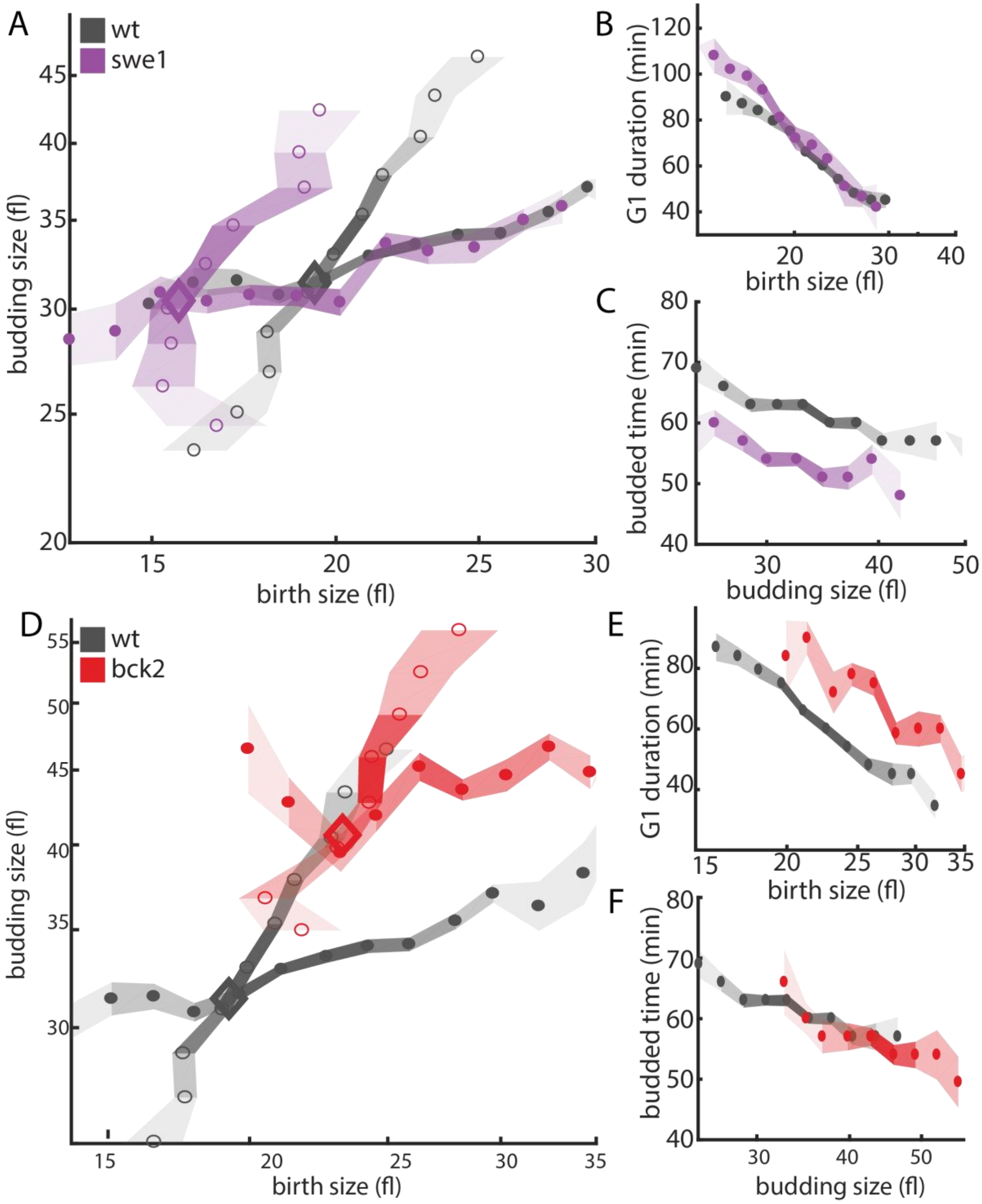

Figure S4: Phase specific effect of BCK2 and SWE1 deletion, related to Figure 6:

A-C) *Cell cycle dynamics after SWE1 deletion*: Size mapping (A), birth size-dependent G1 duration (B) and budding size-dependent budded time (C) of SWE1-deficient cells.

D-F) *Cell cycle dynamics after BCK2 deletion*: Size mapping (D), birth size-dependent G1 duration (E) and budding size-dependent budded time (F) of BCK2-deficient cells.

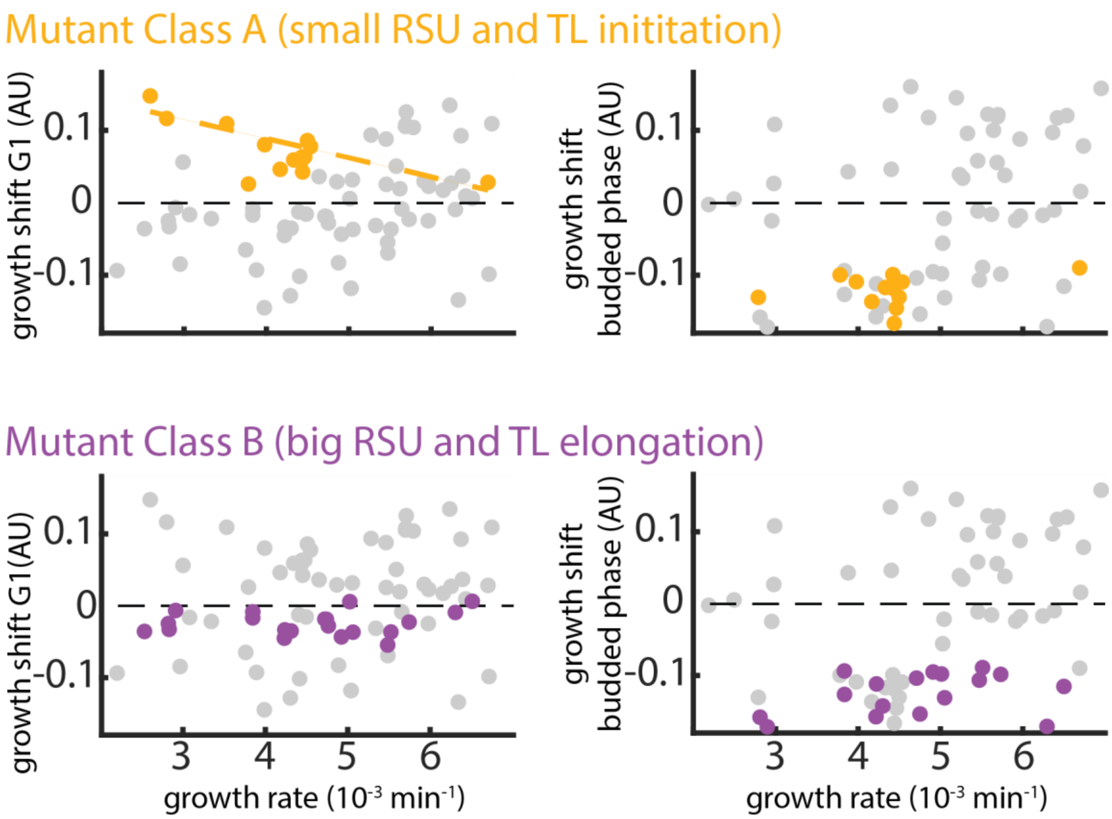

Figure S5: Growth rate dependent growth shift in Class A mutants, related to Figure 5:

A,B) Growth shift in G1 of Class A mutants scales with growth defect. Growth shift of class A mutants, e.g. small ribosomal SU, TL initation, (yellow) and other mutants (grey) in G1 (A) and the budded phase (B).

C,D) *No scaling for growth shift in Class B mutants.* Same as A,B for class B mutants (purple), e.g. big ribosomal SU and TL elongation
